# Prevalence of Hepatitis B and C Infections and HCV Genotypes Among Haemophilia Patients in Ahvaz, Southwest Iran

**Published:** 2012-08-30

**Authors:** Mohammad Ali Assarehzadegan, Mehri Ghafourian Boroujerdnia, Khodamorad Zandian

**Affiliations:** 1 Department of Immunology, Faculty of Medicine, Ahvaz Jundishapur University of Medical Sciences, Ahvaz, IR Iran; 2 Department of Immunology, Faculty of Medicine, Thalassemia and Hemoglobinopathy Research Center, Ahvaz Jundishapur University of Medical Sciences, Ahvaz, IR Iran; 3 Thalassemia and Hemoglobinopathy Research Center, Shafa Hospital, Ahvaz Jundishapur University of Medical Sciences, Ahvaz, IR Iran

**Keywords:** Hepatitis B, Hepatitis C, Prevalence, Genotype, Haemophilia A

## Abstract

**Background:**

Transfusion-transmitted hepatitis is the most important cause of transmitted infections by the parenteral route in patients with haemophilia.

**Objectives:**

This study was performed to determine the prevalence of HBV, HCV, and different genotypes of HCV among haemophilia patients in Ahvaz city, southwest Iran.

**Patients and Methods:**

A cross-sectional study was conducted on 87 haemophilia patients referred to the Hemoglobinopathy and Thalassemia research centre during February 2008 to March 2009. Patients, sera were tested for HBsAg and anti-HCV using ELISA and confirmed by PCR (HBV) and RT-PCR (HCV). HCV genotypes were determined with HCV genotype specific primers using HCV genotyping kit.

**Results:**

The overall prevalence rate of HBsAg and anti-HCV were 1.1% (95% CI: 0-3.39) and 54% (95% CI: 43.5-64.4), respectively. Forty two of the anti-HCV patients (89.3%) were also HCV RNA positive. The prevalence of anti-HCV seropositivity was significantly higher (P = 0.0008) among patients who had started to receive transfusions before implementation of blood donor screening. Moreover, the number of transfusion were significantly associated with anti-HCV and HCV RNA positivity (P = 0.0041 and P = 0.023, respectively). The predominant HCV genotype among haemophilia patients in our region was 1a (26/42, 61.9%), although genotypes 1b and 3a were found in 26.1% (11/42) and 11.9% (5/42) of the patients, respectively.

**Conclusions:**

It appears stringent donor selection procedures reduced HCV infection in multi-transfused patients, but it is still serious risk for these subjects.

## 1. Background

Patients with haemophilia and other coagulopathies are at risk of acquiring several viral infections as a result of their need for multiple blood transfusions. Hepatitis B (HBV) and C (HCV) viruses are the most important causes of transmitted infections by the parenteral route in patients with haemophilia.[1][2][3][4] Various studies among multi-transfused haemophilia patients demonstrated a wide range of prevalence of transfusion-transmitted infections. In Iran, the prevalence of HBV infection ranges from 0.7% to 27% [5][6][7][8] and HCV from 15% to 80% [4][5][6][8][9] in these subjects. Ahvaz city, the capital of Khuzestan province, located in the southwest of Iran, a tropical area with an approximate population of 1.4 million (census 2006). Khuzestan has suffered the heaviest damage of all Iranian provinces during a 28-year period including: the Iran-Iraq War (1980-1988), the Gulf War (1990-1991), and the 19-year crisis in Iraq (1990-2009).[10] This geographical location, mass immigration from Iraq, frequent travels to Iraq and neighboring Arabian countries have all affected prevalence of hepatitis infections in Khuzestan province. [10][11] Moreover, some coagulation disorders are important health problems throughout Iran particularly in this region.[5]

## 2. Objectives

Due to the lack of sufficient reported data from our region, the current study, the first of its kind in southwest Iran, was performed to investigate the prevalence of HBV, HCV infections and different genotypes of HCV among haemophilia patients in Ahvaz.

## 3. Patients and Methods

### 3.1. Patients

This cross-sectional study was performed between February 2008 and March 2009 on haemophilia patients referring to Research Centre of Thalassemia and Hemoglobinopathies (RCTH) in Ahvaz city, Southwest Iran. The study was accepted by our institutional review board. A total of 87 the whole blood were collected from the patients, after obtaining an informed consent. Serum samples were separated from the whole blood, aliquated and stored at -20˚C. Demographic data, such as age, duration and number of blood transfusions were obtained from patient records.

### 3.2. Laboratory Assays

All sera were screened using HBsAg and anti-HCV assays with 3-rd generation of immunoenzymatic test (DIA. PRO Diagnostic, Bioprobes srl, Italy). Positive samples were confirmed using DNA polymerase chain reaction (PCR) and nested RT-PCR for HBV and HCV, respectively, by methods already described.[12][13] briefly, all samples were submitted to DNA and RNA extraction, using high pure nucleic acid kits (Roche, Germany) according to the manufacturer’s instructions. HCV RNA was transcribed into cDNA by random primer (Fermentas, Lithuania). The cDNA was targeted using a nested-PCR with specific primers for the conserved sequences in the 5´ non-coding region (5´-NCR) of HCV. HCV genotypes were determined with HCV genotype specific primers by HCV genotype kit, according to the manufacturer’s instructions (Sacace, Italy).

### 3.3. Statistical Analysis

Prevalence and 95% confidence intervals (95% CI) were calculated by SPSS software version 13.0 (SPSS Inc., Chicago, IL). The prevalence of anti HCV and HCV RNA were compared with variables including age, sex, first transfusion before 1996 , starting transfusion in 1996, number of units transfused ( < 100, 100-200, > 200). Data comparisons were performed using the Chi-square test, Fisher’s exact and two-tailed t-test. The differences were considered significant if p < 0.05.

## 4. Results

Eighty seven haemophilia patients were tested. There were 76 (87.4%) males and 11 (12.6%) females; their mean age (± SD) was 21.83 ± 11.9 years (range 2–69 years) (Table 1). Forty seven patients were positive for HCV antibodies, for a prevalence rate of 54% (95% CI: 43.5-64.4) (Table 1). One HBsAg-infected patient (1.1%, 95% CI: 0-3.39) was positive for

HBV DNA. Moreover, HCV RNA was detected in 89.3% (42/47) of the anti-HCV-positive patients. The mean age of the anti-HCV and HCV RNA positive patients was higher than that of negative subjects (P = 0.0008). In addition, the prevalence of anti-HCV seropositivity was significantly higher (P = 0.0008) among patients who had started to receive transfusions before 1996 when serological screening for anti-HCV antibody had been introduced to blood banks in Iran. The prevalence of anti-HCV and HCV RNA was significantly higher in patients who received blood transfusion more than 200 times compared to subjects with blood transfusion less than 100 times (P = 0.0041 and P = 0.023, respectively). No statistically significant difference was seen between anti-HCV or HCV RNA positive and negative patients with respect to sex. The predominant HCV genotype among haemophilia patients in our region was 1a (26/42, 61.9%), though genotypes 1b and 3a were found in 26.1% (11/42) and 11.9% (5/42) of the patients, respectively.

**Table 1:** HCV Infection Among Haemophilia Patients Receiving Multiple Transfusions

**Characteristics**	**Anti-HCV**			**HCV RNA**		
	**Positive**	**Negative**	**P value**	**Positive**	**Negative**	**P value**
Prevalence, No (%)	47 (54)	40 (46)		42 (48.3)	45 (51.7)	
Age			0.001			0.001
Mean ± SD	29.1 ± 10.7	13.3 ± 5.8		27.3 ± 11.9	16.7 ± 9.3	
**Sex**			0.5			0.1
Male	42 (55.3)	34 (44.7)		39(51.3)	37 (84.4)	
Female	5 (45.5)	6 (54.5)		3 (27.3)	8 (72.7)	
**First transfusion, No (%)**			0.001			0.002
Before 1996	47 (87.0)	7 (13.0)		42 (77.7)	12 (22.3)	
Starting in 1996	0	33 (100)		0	33 (100)	
**Number of units transfused, (%)**			0.005			0.03
< 100	0	9 (100)		1 (11.1)	8 (88.9)	
100-200	8 (34.8)	15 (65.2)		10 (43.5)	13 (56.5)	
> 200	39 (70.9)	16 (29.1)		31 (56.4)	24 (43.6)	

## 5. Discussion

Transfusion-transmitted hepatitis is an emerging public health in many parts of the world, mainly in regions where blood screening practices are poor and the prevalence of parenterally transmitted infections among blood donors is high.[14][15] Patients with hemophilia and other coagulopathies have a high risk of acquiring hepatitis C, hepatitis B, and other viral infections because of the large number of blood transfusions and clotting factor concentrates received.[4][8] In this study, we found that the prevalence of anti-HCV and HBsAg were 54% (47/87) and 1.1% (1/87), respectively. A recent study suggested that blood transfusion was the leading risk factor for HCV acquisition in Khuzestan province as 52% of HCV-positive individuals were diagnosed with chronic haemolytic anaemia and received regular blood transfusion.[16] when serologic tests for HBV and HCV became available, blood donor screening began to be performed in most countries. In Iran, mandatory anti-HCV screening was introduced to blood banks in 1996.[10][14]. In our study, the mean age of anti-HCV positive patients was higher than that of negative patients. Furthermore, the results show that 62% of haemophilia patients included in this study had been transfused more than 12 years, and thus had an increased risk of having received blood products which had not been tested for HCV. In the present study, all of the anti-HCV positive patients had a significantly higher frequency of blood transfusion. However, the wide variation, 15 to 80%, [4][5][6][8][9][17][18][19] in the prevalence of anti- HCV among haemophilia patients may suggest the other risk factors to which the patients were exposed, and may be specific to a country or region.[16][19][20][21] Moreover, one of the most common causes of HCV transmission by transfusion is the occurrence of new infections in blood donors. Prevalence of HCV in blood donors among different provinces of Iran has been reported to be 0.3-0.97%.[1][4][16][21] Nevertheless, a recent study on 2376 blood donors in Ahvaz showed that 2.3% of the subjects were positive for anti-HCV[22]. Noteworthy, the most important problem with the studies is the possibility of selection bias and low positive analytical value of anti-HCV detection by ELISA.[1][15] However, in our study anti-HCV positivity was detected in 47 (54%) of the 87 haemophilia patients. This rate is higher than that in the blood donors in our region. Furthermore, the anti-HCV prevalence in our study was lower than that in the multi-transfused patients in some neighboring Arabic countries (66%-79%) [23][24] and other countries such as Pakistan (56%),[19] Brazil (60%) [25] and Netherlands (68%). [19]

The prevalence of anti-HCV positive was higher among males (55.3%) than among females (45.5%), although the difference was not significant (P = 0.5) (Table 1). However, in some previous studies the male preponderance in HCV-infected patients was reported [16][22]. HCV RNA was detected in 89.3% (42/47) of the anti-HCV-positive patients. In a number of studies among various populations, this rate was a wide range between 48-82%.[17][22][26][27] Epidemiological data show that 10–20% of HCV infected patients eliminate infection and HCV-RNA is not detected anymore in their blood.[7][28][29] However, more care should be taken regarding ELISA test limitations, particularly in routine HCV diagnosis, to avoid unnecessary distress in patients, as a result of false-positive HCV-antibody detection. Studies from some neighboring Arabic countries reported an HCV infection rate of 29.4% to 79% among multi-transfused patients.[24][30][31] Khuzestan province shares a land, river and sea border with these countries. In addition, the Iran-Iraq War of 1980-1988, has had a devastating impact on public health. Moreover, during a period of 18 years, due to poor security and living conditions, many Iraqi refugees have crossed over the Iraqi border to Iran, mainly to the south western regions.[10][11] The geographical situation, mass immigration from Iraq, where a significantly higher prevalence of anti-HCV has been found among various populations [23][30] and frequent travels between Khuzestan province to neighboring Arabic countries, all could affect the prevalence of transfusion-transmitted infections in our region. In our study, HBsAg positivity and HBV DNA was found to be 1.1% among the haemophilia patients. This rate was less than that among blood donors in Iran (1.8% vs. 3%)[2]. This relatively low frequency may be due to vaccination against HBV for newborn and particularly high-risk groups since 1992 and the compulsory screening of donated bloods by the local blood banks since 1995[2][32]. Moreover, Seropositivity for HBsAg in the current study was lower than what other studies showed to be 2.4% to 26.7%.[8][33][34] However, it appears that the rate of HBV infection in total population particularly multi-transfused patients has been declining in Iran since 1995.[19] This study is the first report on prevalence of HCV genotypes among haemophilia patients in Khuzestan province. According to previous studies [12][22][35] among Iranian multi-transfused patients, three HCV genotypes were mainly identified in haemophilia patients in Ahvaz: subtypes 1a, 1b and 3a. Subtype 1a was predominant (60%) similar to the recent studies in various populations in our region.[12][22]. This finding is different with HCV genotypes distribution and the high frequency of genotype HCV 1a/1b plus HCV 4 and HCV 2/2a plus HCV 4 in multi-transfused patients living in neighboring countries, such as Bahrain and Saudi Arabia respectively, sharing sea boarder with Khuzestan province.[36]. In conclusion, this study provides information that will assist in developing intervention guidelines to reduce the risk of acquiring transfusion-transmitted infections which continue to be a significant public health problem in Khuzestan province. The prevalence of HCV infection decreased after introduction of screening tests and stringent donor selection procedures, but these infections particularly HCV in patients with haemophilia are still serious risks for these patients.
